# Non-Invasive Sampling of Schistosomes from Humans Requires Correcting for Family Structure

**DOI:** 10.1371/journal.pntd.0002456

**Published:** 2013-09-19

**Authors:** Michelle L. Steinauer, Mark R. Christie, Michael S. Blouin, Lelo E. Agola, Ibrahim N. Mwangi, Geoffrey M. Maina, Martin W. Mutuku, Joseph M. Kinuthia, Gerald M. Mkoji, Eric S. Loker

**Affiliations:** 1 College of Osteopathic Medicine of the Pacific Northwest, Western University of Health Sciences, Lebanon, Oregon, United States of America; 2 Department of Zoology, Oregon State University, Corvallis, Oregon, United States of America; 3 Centre for Biotechnology Research and Development, Kenya Medical Research Institute, Nairobi, Kenya; 4 Department of Biology, University of New Mexico, Albuquerque, New Mexico, United States of America; James Cook University, Australia

## Abstract

For ethical and logistical reasons, population-genetic studies of parasites often rely on the non-invasive sampling of offspring shed from their definitive hosts. However, if the sampled offspring are naturally derived from a small number of parents, then the strong family structure can result in biased population-level estimates of genetic parameters, particularly if reproductive output is skewed. Here, we document and correct for the strong family structure present within schistosome offspring (miracidia) that were collected non-invasively from humans in western Kenya. By genotyping 2,424 miracidia from 12 patients at 12 microsatellite loci and using a sibship clustering program, we found that the samples contained large numbers of siblings. Furthermore, reproductive success of the breeding schistosomes was skewed, creating differential representation of each family in the offspring pool. After removing the family structure with an iterative jacknifing procedure, we demonstrated that the presence of relatives led to inflated estimates of genetic differentiation and linkage disequilibrium, and downwardly-biased estimates of inbreeding coefficients (F_IS_). For example, correcting for family structure yielded estimates of F_ST_ among patients that were 27 times lower than estimates from the uncorrected samples. These biased estimates would cause one to draw false conclusions regarding these parameters in the adult population. We also found from our analyses that estimates of the number of full sibling families and other genetic parameters of samples of miracidia were highly intercorrelated but are not correlated with estimates of worm burden obtained via egg counting (Kato-Katz). Whether genetic methods or the traditional Kato-Katz estimator provide a better estimate of actual number of adult worms remains to be seen. This study illustrates that family structure must be explicitly accounted for when using offspring samples to estimate the genetic parameters of adult parasite populations.

## Author Summary

Genetic epidemiology uses genetic data to uncover patterns of disease processes. To acquire data for these analyses, individual pathogens are collected and scored at genetic markers, and the resultant data are analyzed to infer biological patterns about the pathogen populations. In lieu of invasive sampling of adult pathogens in humans, researchers have relied on non-invasive sampling of parasite offspring (often shed in fecal samples). One potential problem with this approach is that analyses using the offspring data will be biased because many of the offspring are related and family sizes are likely to be unequal. We show that this sampling issue is relevant in a natural transmission zone in western Kenya and that it yields biases in three important parameters: genetic differentiation, inbreeding coefficients, and estimates of the amount of non-random association between loci (linkage disequilibrium). We also develop a method to remove these biases by removing the sibling structure present in the dataset. Finally, we suggest that our measure of family number, as well as other genetic measures, may be useful measures of the worm burdens in patients.

## Introduction

Infectious disease research is rapidly adopting the tools of evolutionary biology and molecular ecology [1]–[5]. Molecular genetic data, evolutionary theory, and population genetic tools can provide methodology to uncover epidemiological processes that cannot be easily determined otherwise. Such processes include pathogen migration and gene-flow, strain divergence, and selection [6]–[9]. However, some pathogens can be difficult subjects for molecular studies because their adult stages cannot be ethically or pragmatically collected from their human hosts. Thus, researchers often rely on the collection of progeny to infer information about the adult population. Schistosome parasites are one such example. Schistosomes are dioecious blood flukes that become reproductively mature in the vasculature (mesenteric veins or the veins of the bladder plexus) of their hosts where they reside in primarily monogamous pairs [10], [11]. The adults are inaccessible, but their offspring can be collected as eggs that are shed in urine or feces. Consequently, schistosome offspring are often used as a proxy for the adult population, typically to infer worm burdens, and genetic structure among host individuals, host species, and geographic locations [12]–[20].

One challenge associated with using samples of offspring to assay genetic structure is that a sample of offspring may be misrepresentative of the adult population, and can thus give biased estimates of parameters of the adult population [9], [21], [22]. Two types of biased parameter estimates can result when offspring are produced by a small effective number of breeders, *N_b_* (either because few adults were breeding and/or because those that did breed had highly skewed reproductive output). First, the sampling variance in population allele frequencies that arises from sampling the offspring of a small effective number of adult breeders will yield inflated estimates of genetic differentiation among hosts. Second, the strong sibling structure that will exist in a large sample of offspring having a small *N_b_* will cause negative deviations from Hardy-Weinberg equilibrium (i.e. downwardly biased estimates of *F_IS_*) [e.g. 23], [24], [25] and inflated estimates of linkage disequilibrium (LD) among loci within hosts. The likelihood that these sampling artifacts will arise when sampling offspring depends on the number of breeding adults per host, the reproductive skew among those adults, and on the sample size of offspring collected. Furthermore, because it is relatively easy to collect large numbers of offspring, one can reach false conclusions with high statistical confidence.

The distribution of reproductive output among individuals in natural populations of organisms is usually highly skewed, causing the ratio of the variance of reproductive output to the mean reproductive output, 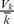 (variance to mean ratio, VMR), of the adults in a population to be much greater than 1 [e.g. Table 10.2 in 26]. In laboratory infections of mice, schistosomes showed VMR ranging from 7.2 to 7.4 [10]. This reproductive skew produced a ratio of effective number of breeders (N_b_) to census number of breeding adults (N_c_) of 0.24. VMR has not previously been measured in natural populations of schistosomes. Therefore, in order to accurately measure important epidemiological parameters, it is essential to determine how large of an effect the above sampling issue will have on population genetic studies of this parasite.

Schistosomes are a substantial public health issue in tropical and developing countries. They are estimated to infect over 200 million people (approximately 1 out of 35) worldwide [27]. Schistosomiasis is a chronic and debilitating disease with a life cycle that is difficult to control. Long-lived and recurrent infections present an ongoing inflammatory challenge that can result in anemia, severe portal hypertension, malnutrition, poor growth, impaired cognitive development, increased suseptibility to coinfection, and increased pathology in coinfection [28]. The schistosome life cycle involves a snail intermediate host and a mammalian definitive host. Eggs are released with the urine or feces of the mammalian host, hatch in water, and release free-swimming miracidia. Miracidia infect snails and undergo asexual reproduction resulting in thousands of clonal cercariae that emerge from the snail daily. Cercariae penetrate the skin of their definitive host and establish long lived infections averaging 6–11 years [29]. Estimating population genetic parameters such as F-statistics of adult schistosome populations is important because they can reveal local transmission patterns and the distribution of genetic variation within and among hosts and geographic regions. Genetic data might also be useful in providing measurements of worm burdens and their effective population size, parameters that can be difficult to measure otherwise [30], [31]. Accurate estimates of these population genetic parameters are sorely needed for effectively targeting drug treatment efforts against schistosomes [32] and to ameliorate reduced drug susceptibility in schistosome populations, which has already been detected in a natural population [33].

The primary aim of this study was to investigate whether sampling artifacts are likely to influence population genetic studies of schistosomes from humans when offspring are sampled in lieu of adults. We collected data from 2,424 miracidia noninvasively sampled from 12 humans in western Kenya. To determine the amount of reproductive skew and family structure naturally present in samples collected from humans, we used microsatellite genotype data to cluster offspring into putative sibships. We investigated how family structure influenced LD, inbreeding coefficients (F_IS_) within hosts, and genetic differentiation (F_ST_ and G_ST_) among hosts, and we developed a correction procedure to remove the bias introduced by family structure. We also investigated whether a single fecal sample taken from a human host would give an adequate representation of the genetic composition of worms in that host, or whether multiple fecal samples should be obtained from a host over several days.

## Materials and Methods

### Empirical Data: Sample Collection and Genotyping

Throughout the paper we use “infrapopulation” to refer to the *adult* worms in a host (patient), and “component population” to refer to all the adult worms in all the hosts of a host population [34]. The term “sample” refers to a sample of miracidia from a host (i.e. the offspring sampled from an infrapopulation). Initially, we measured population genetic parameters of adult and offspring schistosomes that were collected from mice as part of a prior study [10]. Because we detected the predicted biases in these samples from which both offspring and adult populations could be assayed (See Online Supporting Information, S1), we collected data from humans naturally infected with schistosomes so that we could determine if these sampling artifacts are relevant to samples from humans residing in a natural transmission zone.

We obtained miracidia from human fecal samples from twelve participants enrolled in a longitudinal study [35]–[38]. As these samples were considered discarded medical waste they were viewed as “exempt” by the University of New Mexico Internal Review Board. As part of the longitudinal study, patients were monitored periodically, and if infected, were treated with praziquantel. Patients were adult males who work in Lake Victoria near Kisumu, Kenya, and were either car washers or sand harvesters. Car washers stand knee to ankle deep in the lake as they wash vehicles near the edge of the lake. Sand harvesters stand up to chest-deep, shoveling sand from the bottom of the lake into boats to sell to concrete manufacturers. Both groups of men were exposed to schistosome cercariae as they worked. These patients presumably were exposed to the same pool of cercariae in the lake (spatially and temporally), such that we expect no spatial or temporal genetic subdivision among worm populations from different patients. In support of that expectation, no spatial genetic subdivision was found when cercariae were sampled from snails in this same region, nor was there evidence for LD or deviations from HWE in those cercarial samples [39]. Note that those cercarial samples were scored using the same microsatellite loci as used in this study [39].

To obtain miracidia, eggs were hatched using standard protocols [18]. The miracidia were lysed individually in the wells of a 96 well plate and genotyped at 12 microsatellite loci as described by Steinauer et al. [40]. GenBank accession numbers of the loci include the following: AF325695, AF202965, AF202966, AF202968, L46951, AF325698, AF325694 (Multiplex panel P17) and M85305, R95529, AI395184, BF936409, AI067617 (multiplex panel P22). Only those individuals having data for at least 10 of the 12 loci were included in the analysis.

### Detecting Family Structure with Sibship Reconstruction

We used sibship analyses to determine whether family structure was present in schistosomes collected from naturally infected human hosts. COLONY v.2.0 [41], [42] was used to partition individual miracidia into their probable sibling groups. COLONY implements a maximum likelihood approach that can incorporate genotyping errors to identify full-sibling and half-sibling families [42]. Using COLONY, we performed analyses with two different user-defined options, first using a monogamous mating system, and second using a polygamous mating system (to include half sibships). Because there was no empirical support for the presence of half sibling families (see below) and designating a polygamous mating system resulted in suboptimal full sibling family partitions, we used only the COLONY results where monogamous mating was specified (see online supplement S2). Nevertheless, the overall results were very similar when using the samples generated with a polygamous mating system (see Online Supporting Information, S2, for details on the performance of COLONY on these samples). Analyses with COLONY were run with full likelihood, with no priors or known allele frequencies, and one short run per dataset. Our sibling partitions from COLONY were similar to those derived from alternative software packages (see online supplement S3).

The results from COLONY were first used to calculate the number of families occurring in each offspring sample (FSF = “full sib families”). This number included families with a sibship size of 1. Because the total number of families identified in a sample should increase with the number of sampled miracidia, *n*, we also calculated FSF/*n* in order to compare among samples. This number gives a relative estimate of infrapopulation size. We employed a commonly used metric, the variance to mean ratio (VMR) for reproductive success, to quantify the amount of family structure in each sample of offspring. Ratios greater than one indicate a skewed distribution in reproductive output or a large variance in family sizes. It is important to point out that this metric will be downwardly biased when the sample size is much smaller than the true number of breeders, thus it is a minimum estimate. We also estimated the effective number of breeders [N_b_; 43] within each patient as another relative measure of infrapopulation size using the Linkage Disequilibrium (LDNE) and Sibship Assignment (SA) [44] methods. The former was calculated using LDNE [45], and the latter with COLONY v. 2.

### Removing Family Structure—“Correcting Samples”

To correct both empirical and simulated samples for family structure, we randomly sampled one individual per family to create a reduced data set that no longer contained any full-sib individuals. We have named this the “one-per-family” approach. Because there is some inherent stochasticity associated with randomly selecting a single individual from each family, we used custom scripts to automate the process, which allowed us to create a large number of “one-per-family” samples for calculations of genetic parameters. By creating a large number of samples, we can effectively sample all single individuals from a given family and capture the associated mean and variance (See online supplement S4 for a discussion of the variance in these samples).

We predicted that small N_b_ and large family structure within samples of miracidia would increase LD among loci, cause negative deviations from HWE (more negative F_IS_), and inflate F_ST_ among hosts [9]. Therefore, these parameters were compared between the raw, uncorrected samples and the corrected samples from humans. To account for the reduction in sample size in the corrected samples after removing full siblings, we calculated Weir and Cockerham's estimation of F_ST_ (theta), which is unbiased with respect to sample size [46]. We also calculated standardized F_ST_ (F_ST_ of the sample relative to the maximum F_ST_ value possible given the dataset) [47] using RecodeData v. 0.1 [48]. To calculate pair-wise theta, we used the Geneclust package in R [49], [50]. We automated the process to iteratively (1) create a one-per-family dataset for each patient and (2) calculate all pair-wise F_ST_ values between patients. This process was repeated 1000 times, after which we calculated the mean and 95% CIs for the one-per-family F_ST_ values and compared them to the uncorrected data set. We repeated the above procedure to calculate a global (as opposed to pair-wise) value of Weir and Cockerham's F_ST_ using the R package Hierfstat [51]. We also calculated the corrected standardized G_ST_ (G″_ST_) [52] using GENODIVE [53]. G_ST_ is often used as an analog of F_ST_ because F_ST_ is dependent on within sample diversity. For assessment of our correction method, G″_ST_ was also calculated in 10 randomly generated corrected datasets for comparison and tested for significance in each dataset using 10,000 permutations of the data. To calculate within-patient F_IS_ for uncorrected (raw) and 1000 one-per-family samples, we used R scripts to combine observed and expected heterozygosities using the standard equation F_IS_ = (He-Ho)/He [54].

We next exported 50 one-per-family data sets from R and imported them into GENEPOP to test for genotypic disequilibrium (a proxy for LD). For each patient we used 1000 batches and 10000 iterations per batch to calculate the percentage of loci pairs that were significantly associated with one another (p≤0.05, averaged over the 50 one-per-family data sets). Because statistical tests can be affected by sample size, we repeated the above procedure on “downsampled” empirical data sets, where we reduced the sample size of miracidia from each patient to the equivalent sample size in the one-per-family data sets from that patient. Unlike in the one-per-family data sets, in the downsampled datasets we removed individuals *randomly* without respect to the predetermined family structure. This process allowed for equitable comparisons of LD between the one-per-family data sets and the uncorrected (empirical) data sets with the same sample size.

To further validate the one-per-family approach to correcting for family structure, we also created simulated data sets where we could precisely control which individuals belonged to given families. We used a distribution of family sizes that matched the empirical distribution from the COLONY output to recreate the observed family structure. To create simulated adult schistosomes, we used the empirical allele frequencies to generate multilocus male and female genotypes in accordance with HWE. We next paired adults monogamously and randomly selected one allele from each parent to create offspring in accordance with Mendelian expectation. We generated 1000 offspring per adult pair and then randomly sampled a precise number of offspring per full-sib family in accordance with the COLONY output from the empirical data for family size. For example, if a patient had two families of sizes 5 and 30, then 5 full-sib offspring would be sampled from one pair, 30 full-sib offspring would be sampled from the next pair, and the remaining offspring would be discarded. Each offspring was assigned a unique individual and family ID to validate the downstream analyses. All simulated data sets were constructed with a script written in R 2.15.1 [55]. A fully annotated version of this script is freely available at dryad. Using the exact approach as described for the empirical data, we tested the effect of correcting samples on 1000 simulated datasets for measuring F_ST_, F_IS_, and LD. Notice that with these simulated data sets we knew the family structure with 100% certainty, whereas with the empirical data we assumed that COLONY accurately captured all of the family structure. Thus, these simulated data sets allowed us to accurately verify the utility of our approach to correcting for family structure.

As even further proof of principle, we also conducted analyses with Kenyan schistosomes in a mouse model system [two mice infected with field-collected schistosomes; see 10 for laboratory methods]. Because we could sample both the adults and the offspring (which is not logistically feasible with human patients) the samples of offspring could be directly compared to the samples of adults for measurements of linkage disequilibrium, F_ST_, HWE, and parentage analysis (See Online Supporting Information, S1, for details).

### Temporal Samples from a Patient

If reproductive rates are constant, then samples from a single patient should be genetically homogenous over multiple days. However, if reproduction occurs in “bursts”, then sampling a single fecal sample could miss diversity within a patient. For three patients 2–3 fecal samples were collected 1–2 days apart. To determine if these samples differed in genetic composition, we first calculated pairwise F_ST_ between temporal samples from the same patient using the raw data and tested their significance via 10,000 permutations of genotypes among samples using FSTAT 2.9.3 [56]. Standardized F_ST_ (F′_ST_) was calculated and standardized G_ST_ (G″_ST_) was calculated and tested for significance with 1000 permutations using GenAlEx v. 6.5 [57], [58].

Next, for the patients for which the raw data indicated significant temporal differences, we corrected them for family structure using our one-per-family approach. Families were resampled 1000 times and mean pairwise F_ST_ was calculated among sample days within each of the three patients. Because the reduced sample size should influence significance testing, we performed the same tests on downsampled samples to match the sample size of the corrected samples.

### Estimating Worm Burden Using Genetic Measures

Another application of the calculation of the number of full sibling families in a sample of miracidia is to estimate the minimum worm burden within a patient (i.e. minimum number of breeding pairs). However, it is possible that with adequate sampling, this measurement, as well as other genetic parameters, may serve as relative or even absolute measurements of worm burden. The number of families detected, as well as other genetic parameters such as allelic diversity, should be strongly correlated with the number of worms that were reproductively active when the sample was taken. With this aim, we investigated the relationship of several parameters obtained from our genetic data and compared them to the World Health Organization gold-standard estimator of worm burden, the number of eggs per gram of feces as determined by the Kato Katz method [59]. The genetic parameters we measured included: the number of full sibling families (FSF), a standardized measure of full sibling families (FSF/n), allelic richness (number of alleles rarefied to the smallest sample size (AR), and the effective number of breeders [N_b_; 43]. N_b_ was estimated using the sibling assignment method implemented in COLONY [44] and the linkage disequilibrium method implemented in LDNe [45]. Although the actual worm burdens in our patients cannot be determined, it is worth determining the extent to which the genetic estimators are intercorrelated and capture the same information as the Kato Katz method (eggs per gram of feces). Pearson's correlations between egg count (i.e. Kato Katz), and the genetic parameters: FSF/n, AR, and N_b_ were performed using Graphpad Prism v. 5.01 (GraphPad Software, Inc.). To further determine similarity among the estimators, a multivariate principal components analysis (PCA) using egg output (i.e., Kato Katz), FSF/n, AR, and both measures of N_b_ as variables was performed using Systat 11 (Systat Software, Inc.).

## Results

### Sibship Analysis

**Figure 1:**
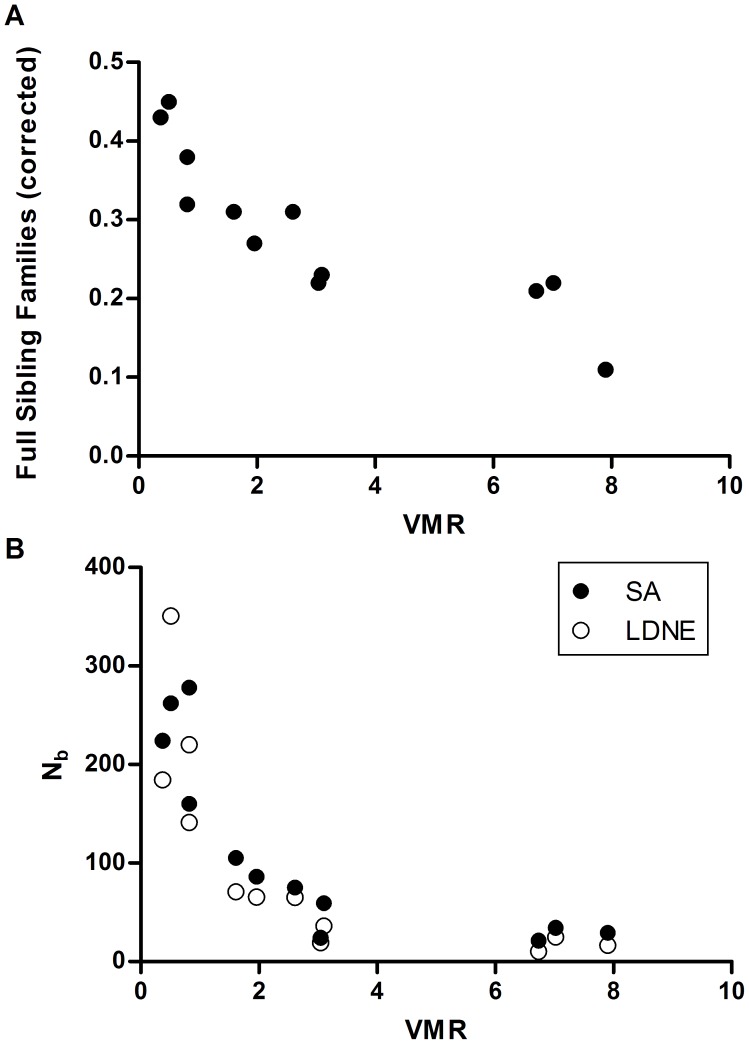
Relationship between the amount of family structure as quantified by the variance to mean ratio of family size (VMR) in samples of *Schistosoma mansoni* offspring collected from 12 patients and A. the estimation of the number of full sibling families (corrected for sample size) as determined by sibship analysis and B. The effective number of breeders N_b_ calculated using the sibling assignment method (SA) or the linkage disequilibrium method (LDNE).

The samples of miracidia from humans varied substantially in the amount of family structure they contained (Table 1, Figure 1). The percentage of miracidia that belonged to a family of four or more (i.e., the number of miracidia which belong to a family with robust support) ranged from 18–91% among samples, and the variance to mean ratio (VMR) for the number of offspring per family ranged from 0.37 to 7.9. The lower values of VMR likely reflect our inability to detect a skew in samples with very large populations rather than equal reproductive output among families. When infrapopulations are large, one needs very large sample sizes to accurately describe the family-size distribution (else you wind up with mostly unrelated individuals). Indeed, several lines of evidence in our data indicate that samples with measured VMR less than one are likely those in which the sample size is much smaller than the actual number of families. First, the ratio of the estimated N_b_ to the sample size was high in samples with VMR<1 (Table 1, Figure 1). Furthermore, the percentage of individuals belonging to a family of 4 or more was highly correlated with VMR (Pearson's r = 0.807, P = 0.0008) indicating that the samples with a low VMR had a large number of families rather than having a small number of families of equal size. Thus, the measured reproductive skew of samples will be highly dependent on the infrapopulation size and the sample size.

**Table 1:** Descriptive statistics for 12 infrapopulations of *Schistosoma mansoni* derived from human patients in Kenya.

Patient	1	2+	3	4	5	6	7^+^	8^+^	9	10	11	12
**Eggs/g**	60	60	366.7	300	390	110	240	160	220	60	520	116.7
**n**	190	231	412	201	205	203	186	81	192	110	178	235
**OPF n**	82	103	133	76	64	54	57	18	44	23	40	26
**AR**	140.8	148.9	141.0	145.4	141.0	134.1	132.1	121.5	135.6	114.5	127.4	122.4
**VMR**	0.37	0.51	0.82	0.82	1.61	1.96	2.61	3.04	3.10	6.73	7.02	7.90
**FSF**	82	103	133	76	64	54	57	18	44	23	40	26
**Largest FSF**	5	7	10	8	13	14	18	12	15	22	36	36
**%**	18	19	49	35	52	63	54	75	82	75	75	91
**FSF/n**	0.43	0.45	0.32	0.38	0.31	0.27	0.31	0.22	0.23	0.21	0.22	0.11
**N_b_ SA**	224 (179–283)	262 (213–328)	278 (231–235)	160 (124–208)	105 (79–141)	86 (65–117)	75 (54–104)	24 (15–42)	59 (42–84)	21 (13–42)	34 (22–53)	29 (18–47)
**N_b_ LDNe**	185 (143–251)	351 (249–555)	220 (186–263)	141 (112–184)	70 (61–83)	66 (57–75)	65 (56–77)	19 (16–22)	36 (32–41)	10 (9–12)	25 (22–28)	16 (15–18)

+ = HIV positive, n = number of miracidia sampled, OPF n = number of miracidia sampled in the one-per-family correction, AR = allelic richness rarified, VMR = variance to mean ratio of family size, FSF = number of full sibling families, % = the percent of miracidia belonging to a family of 4 or greater, N_b_SA = effective number of breeders N_b_ estimated by the sibling assignment method, N_b_LDNe = effective number of breeders N_b_ estimated by LDNe.

In the analyses of samples of miracidia from humans in which the mating system was designated as “polygamous”, many half sibships were inferred in the COLONY partitions. However, most of these included only 2 or 3 members, or involved a large full sibling group with one or two half siblings. Such small groupings are likely to be spurious. Only three patients had half sibling families that consisted of greater than three individuals per FSF (two such families per patient). Furthermore, analysis of our simulated samples revealed that the majority of half-sib assignments were incorrect (see Online Supporting Information, S2). Thus, we do not see strong evidence for a large number of half sibling groups in our samples from humans, which indicates that these patients were not getting infected with large numbers of genetic clones derived from a single snail.

### Detecting Sampling Artifacts

**Figure 2:**
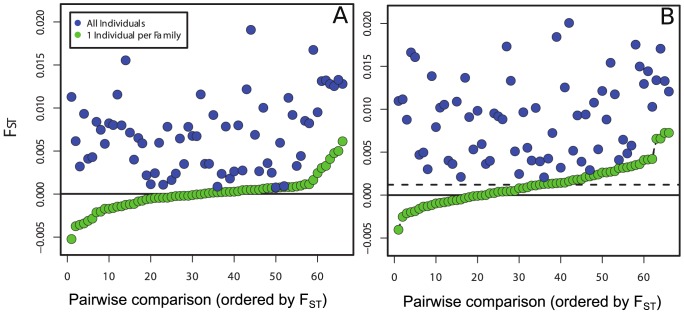
Calculations of pairwise F_ST_ between A. simulated and B. empirical schistosome infrapopulations of 12 human patients as measured by sampling schistosome offspring rather than adults. Both plots show the pairwise comparisons of the raw, uncorrected samples (blue) and those of the same samples corrected using the one-per-family method described in the manuscript (green). Pairwise comparisons are ordered by F_ST_ value on the X-axis. Note that in both plots, the F_ST_ values of the corrected dataset are lower (or equal to) the F_ST_ values from the raw samples showing the predicted inflation caused by family structure in the raw samples. Also note the mean F_ST_ indicated by the dashed line is slightly greater than 0 for the empirical samples, which suggests that a small amount of residual F_ST_ was not removed by the correction. Whether this represents true F_ST_ among patients or a failure of COLONY to accurately identify all sibship is unknown.

The global F_ST_ for the uncorrected data set was 27.8 times higher than the corrected, one-per-family data sets (Corrected dataset global F_ST_ = 0.00026; Uncorrected dataset F_ST_ = 0.0074; Uncorrected dataset standardized global F′_ST_ = 0.027). G″_ST_ indicated significant population subdivision of the uncorrected dataset (G″_ST_ = 0.031, P = 0.001), and was greatly reduced in the 10 corrected datasets and permutation tests indicated no significant population subdivision (mean G″_ST_ = −0.0013, range −0.004 to 0). Thus, correction of the dataset reduced standardized G″_ST_ from 0.031 to a mean of −0.0013; and statistical significance was lost with this correction. Furthermore, pairwise F_ST_ between patients was greatly reduced in the one-per-family data sets (Figure 2). In fact, for the simulated datasets the mean pair-wise F_ST_ was reduced to 0. As expected, this analysis revealed that Weir and Cockerham's F_ST_ estimator is unbiased, though some comparisons differed from 0 due to decreased precision. The analysis using the empirical data set yielded a nearly identical result, with most of the genetic differentiation being removed from the corrected data sets. Interestingly, the mean pairwise F_ST_ for the empirical data set was slightly greater than 0 (approximately 0.001), suggesting a very low level of residual real or artifactual differentiation (see Discussion). The one-per-family correction worked well for both the empirical and simulated data and the confidence intervals surrounding the means from the 1000 iterations were narrow (smaller than the points on the plots). Also, it is important to note that simply downsampling the data randomly (without regard to family structure) to match the sample sizes of the corrected data yielded a mean pairwise F_ST_ value (from 5000 resampled datasets) that was very similar to the value for the uncorrected data (F_ST_ = 0.009). Thus, the change in F_ST_ value was due to the removal of family structure.

**Figure 3:**
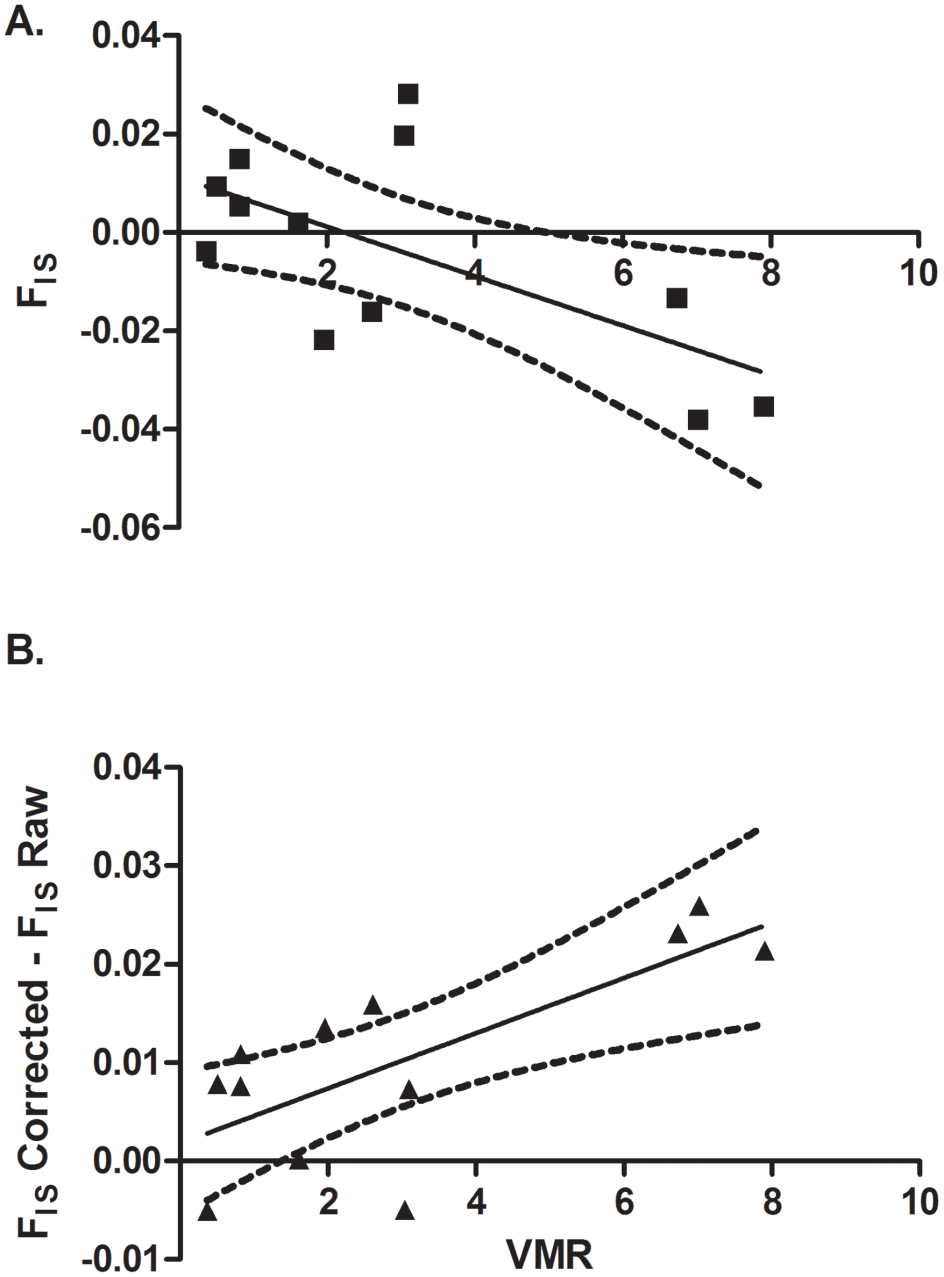
(A). The relationship between F_IS_ and the amount of family structure [measured by the variance to mean ratio of family size (VMR)] among samples of schistosome offspring collected from 12 patients. The negative relationship is as expected because family structure tends to cause heterozygote excess. (**B**) Change in *F_IS_* after correcting each dataset for family structure. Note that in all but two samples the corrected estimate of *F_IS_* is more positive than the uncorrected estimate. Regression lines and the 95% confidence intervals are given for each plot.

As predicted, samples with a large amount of family structure also yielded lower estimates of F_IS_ as indicated by the negative correlation between F_IS_ and VMR (r = −0.638, P = 0.0128) (Figure 3A). This relationship was not detected in the corrected samples (r = −0.3797, P = 0.112). The increase in F_IS_ between raw and corrected samples was greatest in the samples having most family structure and these values were positively correlated (r = 0.733, P = 0.003) (Figure 3B).

**Figure 4:**
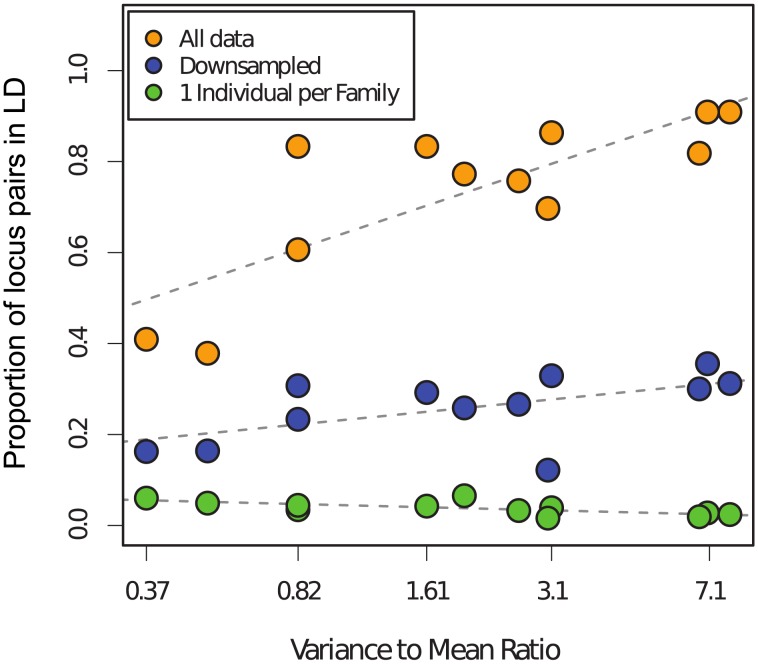
Linkage disequilibrium (LD) between pairs of microsatellite loci within samples of schistosome offspring collected from 12 human patients compared to the amount of family structure present in each dataset. Amount of LD is represented by the proportion of locus pairs in disequilibrium (see text for statistical tests). Family structure is represented by the log-transformed variance to mean ratio of family size (VMR), but plotted on their actual values. LD is shown for each raw dataset (orange), samples corrected for family structure using the one-per-family approach (see text) (green), and the raw samples that were resampled to equal the sample size of the corrected samples (blue). Note the positive relationship between LD and VMR in the raw samples and the near-complete reduction in the corrected samples.

All of the uncorrected samples of miracidia from humans showed significant genotypic disequilibrium, ranging from 38 to 91% of pairwise comparisons of loci (Figure 4). This percentage of loci was much lower in the corrected samples ranging from 0.015 to 0.065%, suggesting that most of the linkage disequilibrium was due to family structure. Correction also reduced the number of loci in LD when compared to the raw samples that were downsampled to match the same sample size as the corrected samples (Fig. 4). Correction also removed the relationship between VMR and the percentage of loci in disequilibrium.

The same sampling artifacts were detected in the samples from mice (See Online Supporting Information, S1, for details and methods). F_ST_ between the adult schistosome infrapopulations in the two mice was low and not statistically significantly different from zero (−0.026 [95% CI: −0.032–0.01]; P = 0.998). In contrast, F_ST_ was substantially higher (0.021 [95% CI: 0.014–0.029] and statistically significant between the samples of miracidia collected from each of the mice (P = 0.0001). Although, neither adult nor offspring samples deviated significantly from HWE, point estimates of F_IS_ were more negative in the samples of miracidia, consistent with predictions. These samples of miracidia also showed many pairs of loci in significant linkage disequilibrium, while the adult samples showed no loci in significant disequilibrium.

### Temporal Samples from a Patient

When the temporal fecal samples from the same patient were analyzed in their raw form (no correction for sibships), allele frequencies of miracidia differed significantly among fecal samples collected on different days for two of the three patients (patient 2: F′_ST_ = 0.021, P = 0.0009, G″_ST_ = 0.021, P = 0.003; patient 3: F′_ST_ = 0.001, P = 0.333, G″_ST_ = 0, P = 0.513; patient 12: F′_ST_ = 0.020, P = 0.0001, G″_ST_ = 0.018, P = 0.002). We sampled patient 12 on three days. Pairwise tests indicated that the fecal sample collected from day 3 was significantly different from those on day 1 and day 2, but samples from day 1 and 2 were not significantly different (Day 1&2: G″_ST_ = 0.006, P = 0.173; Day 1&3: G″_ST_ = 0.028, P = 0.001; Day 2&3: G″_ST_ = 0.019, P = 0.003).

We corrected the samples using the one-per-family method only for those two patients for which the raw data showed significant differentiation from the pairwise F_ST_ tests. Correcting the samples reduced global F_ST_ in all the patient samples and the amount of correction was the highest in the patient with the highest VMR (#12) (patient 2: raw/corrected F_ST_ = 0.006/0.003; patient 12: 0.006/0.001). After correction, there were no significant temporal differences between fecal samples. However, the differences between the uncorrected samples also became non-significant when they were downsampled to match the sample sizes of the corrected samples.

### Estimating Worm Burden Using Genetic Measures

**Figure 5:**
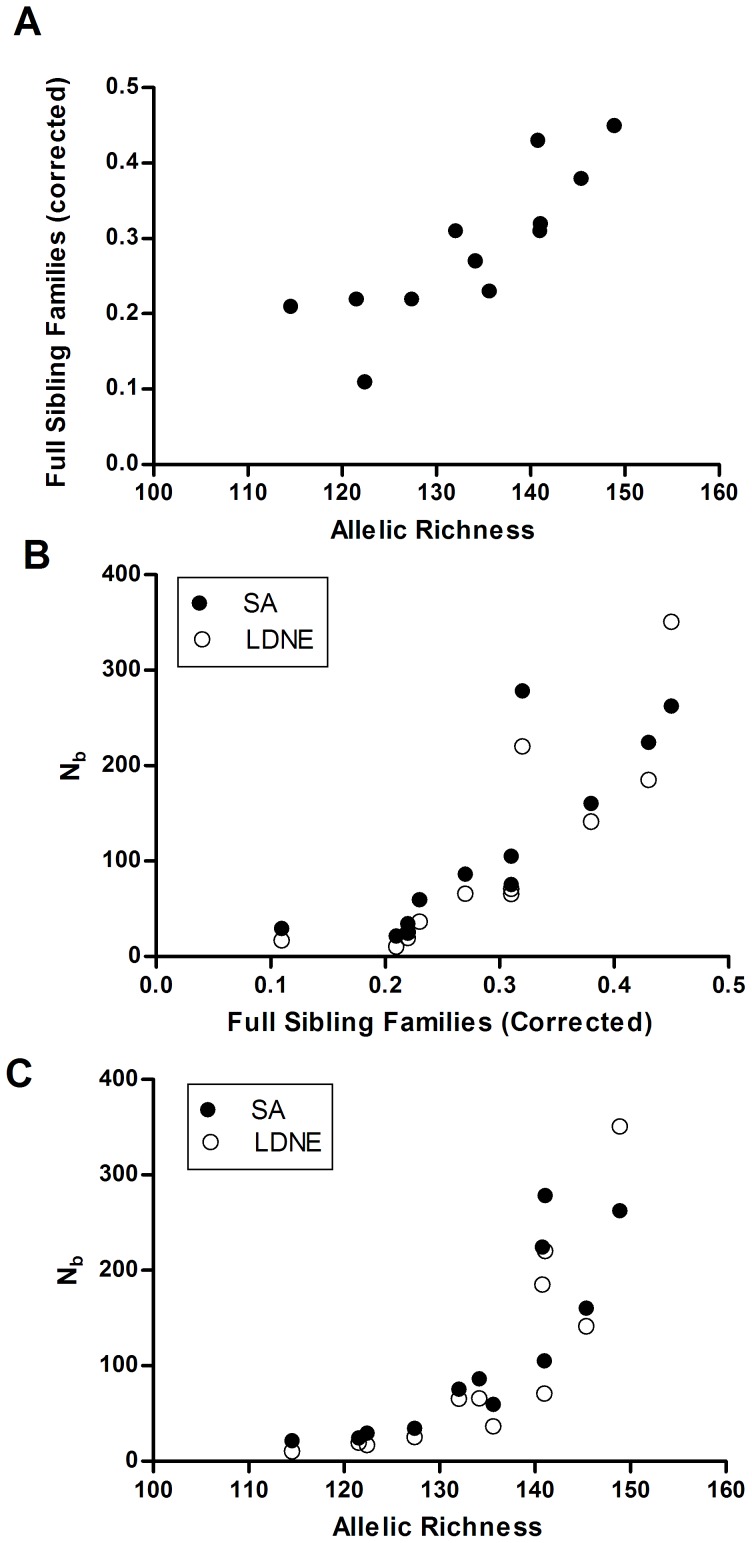
Relationships between A. allelic richness and the standardized number of full sibling families estimated by kinship analysis, B. the standardized number of full sibling families and the effective number of breeders estimated by the sibling assignment method (SA) and the linkage disequilibrium method (LDNE), C. allelic richness and the effective number of breeders.

Egg output (i.e., Kato Katz method) was not correlated with either the number of full sibling families FSF/n (r = −0.097, P = 0.383), with AR (r = 0.153, P = 0.318), or either measure of N_b_ (SA: r = −0.049, P = 0.440; LDNE: r = −0.169, P = 0.300). However, PCA analysis indicated that all the genetic factors were strongly related and loaded heavily on one factor (loading values = 0.90–0.96), with egg counts loading heavily on a second factor (0.99). Factor one (genetic measures) explained 69.1% of the variation and factor two (egg counts) explained 22.2% of the variation. Further examination of univariate correlations indicated that all the genetic measures were strongly correlated (Pearson's r = 0.79–0.83 and P≤0.001) (Fig. 5). The strong intercorrelations among the genetic parameters suggests they are all capturing the same information about variation among patients in number of families contributing to each sample. However, that variation among patients is uncorrelated with variation in the traditional, Kato-Katz estimate of worm burden.

## Discussion

We demonstrated that samples of *S. mansoni* miracidia collected from human hosts can contain large numbers of full-sib families and that not explicitly accounting for this family structure could cause one to draw false conclusions concerning population structure. Importantly, this bias could occur in any system where offspring are sampled in lieu of the adults, and has previously been documented in a variety of systems [e.g. 21], [60], [61], [62]. As predicted, the presence of family structure in samples was correlated with reduced measures of F_IS_ and inflated measures of linkage disequilibrium and F_ST_ values. Correcting for family structure in our samples of miracidia had a large effect on estimates of population genetic parameters. For example, the number of pairs of loci in linkage disequilibrium in the empirical samples was dramatically reduced after correction (from a maximum of 91% to a maximum of 0.065%). Similarly, correcting for family structure yielded global F_ST_ values that were 27 times lower than that from the uncorrected samples. These results were further corroborated by data simulations. F_IS_ values were also downwardly biased by the presence of family structure, although this bias was not large enough to cause the offspring samples to significantly deviate from HWE (data not shown). Even though this bias appears to be small, it could mask important biological information, such as inbreeding or Wahlund effects, or even important laboratory artifacts (e.g., null alleles), that could otherwise be detected. A heterozygote excess over HWE expectations is predicted for samples that contain many siblings and the extent of the excess is increased in hosts having lower N_b_ and higher family structure [23]–[25], [63], [64].

The upward bias measured in global F_ST_ in this study may, at first glance, appear to be modest. However, the documented bias is non-trivial and could result in the misinterpretation of important biological processes. For example, even after Bonferroni corrections, pairwise F_ST_ tests among uncorrected patient infrapopulations (i.e. including siblings) show highly significant population differentiation among all patients. Consequently, both the global and pair-wise estimates of genetic differentiation may cause researchers to erroneously conclude that the samples were genetically differentiated. However, because all patients were exposed to the same pool of cercariae both spatially and temporally, and more importantly, because both the simulated and empirical data sets that were corrected for family structure yielded mean F_ST_ estimates close to zero (0, 0.001), we conclude that there is no real genetic differentiation between patients. This conclusion is further reflected in the data from the mouse system. Even though the adult populations were not differentiated, the uncorrected offspring datasets appeared to be. Thus, biases presented by including siblings in population genetic analyses could potentially affect the interpretation of all of the datasets presented here. More importantly, these biases could affect a wide variety of larger-scale studies that make genetic inferences based on sampling offspring.

Here, we also present a correction method that yields population genetic parameter estimates with this bias removed or greatly reduced. Although some loci remained in LD and the average F_ST_ of the pairwise comparisons of empirical data was marginally positive in the corrected samples, the correction yields much improved estimates that are likely to be more biologically meaningful. We hypothesize that the residual positive F_ST_ and loci in LD are due to family structure not recognized by the sibship analysis and thus not removed from the dataset after correction. This family structure could be due to failure of the sibship analyses to recognize small sibling families or the presence of small half sibling families in the samples (our sibship analyses did not uncover half-sibships with large membership in our samples). Half-sibships could theoretically be derived from clonal cercariae (products of asexual reproduction in the snail) that are transmitted together to the same definitive host and pair with genetically different partners. This mating pattern might occur if the cercariae are unable to move far from their infected snail (e.g. constrained to a small pool). However, our results suggest that most cercariae are well-mixed before infecting their hosts. Another way half-sibships could be produced is through mate switching. However, mate-switching is not likely to occur in our samples because each fecal sample represents a small window of reproduction and schistosomes are primarily monogamous [10], [11], [65].

We have shown that significant family structure is likely to be present in samples of miracidia collected from human patients. Therefore, using uncorrected genetic data collected from schistosome offspring (miracidia) to infer important epidemiological parameters of the adults is likely to generate false conclusions. Failure to correct for family structure can cause one to overestimate F_ST_, linkage disequilibrium, and heterozygosity (negative bias in F_IS_). Previous schistosome epidemiological studies have used a hierarchical AMOVA approach in order to remove the bias caused by family structure in offspring samples [e.g. 12], [66]. Although this method may be successful by removing the among infrapopulation variance and thus bias caused by family structure so that the data may be interpreted at the top hierarchical levels (e.g. geography), this approach does not make corrections for comparisons at the infrapopulation level.

### Temporal Samples from a Patient

We observed a statistically significant F_ST_>0 between the raw data from two sets of temporal samples that were each collected from a patient over multiple days. Tests using only the raw data would cause one to conclude that genetically different subsets of worms are producing eggs on different days. Family structure and differential representation of families in sequential fecal samples appear to be driving the statistical differences in our data. It should be noted that these biases can occur in samples with both large and small family structure (i.e. patient 12 and patient 2, see online supplement, S5). The differentiation between samples may be due to random sampling error (some families are missed by chance), or biological attributes that change a worm pair's contribution to a single fecal sample (i.e. location in host, age, competition among pairs). To answer this question, much larger samples are necessary. In any case, fecal samples from multiple days may be more representative than a single fecal sample. However, it still remains unclear whether sampling artifacts can be best overcome by increasing the sample size on one day, or collecting several small samples over multiple days.

### Estimating Worm Burden Using Genetic Measures

The number of full sibling families detected in a sample of miracidia is a measure of the minimum number of worm pairs present in a patient. The strong covariation among genetic variables in our data suggests that genetic parameters could be used further: to depict relative worm burdens in patients. It is also possible that, given a large enough sample size, the true worm burden within a patient could be detected via the number of full sibling families. The challenge will be obtaining a large enough sample size to account for the true worm burden, a parameter that unfortunately may not be known without obtaining genetic data first. As shown in Table 1, the sample size necessary to detect most sibling families varies widely among patients. For example, for Patient 6, 75% of the miracidia collected were partitioned into robust full sibling families (>3) with a sample size of 81 miracidia. In contrast, with 412 sampled miracidia, only 49% were partitioned into robust families for Patient 3. However, it may be possible to obtain accurate worm burden estimates even without exhaustive sampling, by fitting the observed sibship sizes to an expected sibship size distribution to predict the number of unsampled sibships.

The lack of correlation among fecal egg counts (i.e., Kato Katz) and our genetic proxies for worm burden is interesting, particularly considering the strong correlations detected with genetic measures even with a low sample size of 12 patients. The Kato Katz methodology for egg enumeration is known to be highly variable between fecal samples collected from the same patient and has been deemed unreliable by some particularly when worm burdens are low [67]–[69]. However, others have found evidence of reliability of this method across a broad range of infection intensities [e.g. 70], [71]. It may be that the infection levels in our study were not broad enough for the Kato Katz method to accurately detect relative differences in the worm burdens of our patients. Although we have no independent data to determine which approach gives the most accurate estimates of the true number of adult breeding worms, we suggest that genetic methods potentially give more reliable estimates of the relative number of adult breeding worms per host. These methods should be explored further because they could be valuable tools for epidemiological studies that measure the success of control programs.

A previous study did not find a relationship between their calculated N_e_ from schistosome offspring populations and allelic richness [19]. However, it appears that their estimates of N_e_ may not have been very accurate because there was no correlation between N_e_ values from the same samples calculated by two techniques and they were estimated with large confidence intervals. This lack of accuracy could be due to the small number of markers used, relatively small sample sizes (10–30 miracidia per patient), and pooling of samples from several infrapopulations rather than using a subdivided breeders model [31] to calculate N_e_. The lack of correlation may also be due to saturation of allelic richness since there is a limit to the number of alleles that will be found in a population and a correlation beyond this saturation point is not expected.

### Summary

Genetic epidemiology is a powerful tool for infectious disease research. However, in cases where offspring must be collected in lieu of adults, data analysis and interpretation should be carefully considered. We have shown that samples of parasite larvae collected from humans can contain significant family structure, which can lead to inflated estimates of linkage disequilibrium and F_ST_, and underestimates of F_IS_. The amount of bias in each of these parameters is positively correlated with the skew in reproductive output of individuals. It should also be noted that sibling structure and skewed reproductive output among individuals (small N_b_) could skew additional population genetic parameters and analyses not evaluated here such as observed heterozygosity (H_o_), gene diversity (H_e_) [72], genetic distance, and clustering algorithms, thus, care should be taken in their interpretation. Correcting samples by performing a sibship analysis and then excluding all but one member of each full sibling family is effective at removing or reducing this bias. The number of full sibling families detected by our analyses gives an estimate of the minimum number of worm pairs within a patient and may be a reliable estimator of the relative worm burdens within patients, an important epidemiological parameter.

### Data Accessibility

Microsatellite genotype data and annotated R scripts to perform the “leave-one-out” procedure will be made available at Dryad.

## Supporting Information

Supporting Information S1 Schistosome infections from a model system: Proof of principle.
(DOCX)

Supporting Information S2 Performance of colony using monogamous and polygamous mating system designation.
(DOCX)

Supporting Information S3 Performance of alternative methods of sibship reconstruction.
(DOCX)

Supporting Information S4 Assessing the variance in the “corrected” one-per-family datasets.
(DOCX)

Supporting Information S5 Temporal sampling: Examining the genetic distribution of miracidia obtained from temporally collected fecal samples.
(DOCX)
