# Supplementary material for: Non-Invasive Sampling of Schistosomes from Humans Requires Correcting for Family Structure
Source: PLoS Negl Trop Dis. 2013 Sep 19;7(9):e2456. doi: 10.1371/journal.pntd.0002456 (PMC3777896; doi:10.1371/journal.pntd.0002456)
Supplement: Supporting Information S1 — Schistosome infections from a model system: Proof of principle. (DOCX) [file pntd.0002456.s001.docx]

**Schistosome Infections from a Model System: Proof of Principle**

As additional proof of principle for the sampling bias that we describe in the manuscript, we infected mice with Kenyan schistosomes [see 1 for laboratory methods] and compared estimates of F_ST_, Hardy-Weinberg Equilibrium (HWE), and linkage disequilibrium (LD) obtained directly from the adult schistosomes to those obtained by sampling their offspring. Two mice were infected. Mouse 1 contained 13 mating pairs of schistosomes and mouse 2 contained 9 mating pairs. Totals of 89 and 49 miracidia were sampled from Mouse 1 and 2, respectively.

**Sibship Assignment.** For the mouse datasets with known adult genotypes, PEDIGREE [2] and COLONY v2.0 [3,4] were used for sibship assignment as described above and in the manuscript. The results of these analyses were compared to a parentage analysis that included all the potential parents and the offspring (PARENTAGE v. 1.0; Emery et al., 2001). Sibships reconstructed by PARENTAGE were 100% identical to those reconstructed by sibship clustering. Thus, both COLONY and PEDIGREE gave 100% accurate sibship reconstructions in the mouse datasets.

**Linkage Disequilibrium.** We quantified LD in each sample by performing exact tests for genotypic disequilibrium between pairs of loci within each sample using the default parameters in GENEPOP 4.0 [5]. Corrected samples have smaller sample sizes than uncorrected samples which could influence significance of these statistical tests. Therefore, we randomly resampled the full offspring sample so that for each patient the sample size was equivalent to that of the smallest corrected dataset. The percentage of pairwise combinations of loci that showed highly significant (α estimated at 0) genotypic disequilibrium was used to compare samples. In the offspring datasets, many pairs of loci were in significant linkage disequilibrium (59.1% and 34.9% of pairwise locus comparisons were in disequilibrium in mouse 1 and 2 respectively), while the adult samples showed no loci in significant disequilibrium. However, when the offspring data were resampled to match the sample size of adult genotypes (14 and 11), significance declined to 1.5 and 3 % of loci.

**F_IS_**. Significance of deviations from HWE in samples from each dataset (corrected and uncorrected) were assessed by permuting alleles among individuals within each sample and using Weir and Cockerham’s [6] F_IS_ as the test statistic (FSTAT 2.9.3 [7]; 10,000 permutations). Neither adult nor offspring samples deviated significantly from HWE, although point estimates of F_IS_ were more negative in the miracidial samples, as expected (F_IS_ = -0.015 and -0.012 in adults versus - 0.056 and -0.073 in miracidia).

**F_ST_:** Global F_ST_ [6] among hosts (samples) and 95% confidence intervals were calculated using the bootstrap procedure in FSTAT. Statistical significance of F_ST_ > 0 was assed via 10,000 permutations of genotypes among samples. We also estimated F_ST_ between each pair of samples (between adult samples, between offspring samples, and between adult and offspring samples from the same mouse) and tested each pairwise comparison for statistical significance by permutation tests using FSTAT with significance levels adjusted for multiple comparisons using the sequential Bonferroni procedure. F_ST_ between the adult schistosome infrapopulations in the two mice was low and not statistically significantly different from zero (-0.026; P = 0.998). In contrast, F_ST_ was substantially higher (0.021) and statistically significant between the two miracidial samples (P = 0.0001), analogous to samples that would be collected from human patients. F_ST_ was not significant between the adult and offspring populations from the same mouse (mouse 1: F_ST_ = -0.007, P = 0.969; mouse 2: F_ST_ = -0.015, P = 0.988). Thus we see the predicted strong inflation in estimated F_ST_ from sampling offspring rather than the adults to which we actually want to make inference [8].

In conclusion, two infrapopulations were sampled from a common gene pool that was in HWE and linkage equilibrium. Yet, the two samples appeared to be significantly differentiated, simply as a result of sampling offspring rather than the adult parents that make up the infrapopulations. It is also interesting to note that the offspring populations did not differ significantly from their adult populations thus, using this requirement to suggest biases are avoided is not valid [e.g. 9].

**References**

1. Steinauer ML (2009) The sex lives of parasites: Investigating the mating system and mechanisms of sexual selection of the human pathogen *Schistosoma mansoni*. International Journal for Parasitology 39: 1157-1163.

2. Smith BR, Herbinger CM, Merry HR (2001) Accurate partition of individuals into full-sib families from genetic data without parental information. Genetics 158: 1329-1338.

3. Wang J, Santure AW (2009) Parentage and sibship inference from multilocus genotype data under polygamy. Genetics 181: 1579-1594.

4. Jones OR, Wang JL (2010) COLONY: a program for parentage and sibship inference from multilocus genotype data. Molecular Ecology Resources 10: 551-555.

5. Rousset F (2008) genepop™007: a complete re-implementation of the genepop software for Windows and Linux. Molecular Ecology Resources 8: 103-106.

6. Weir BS, Cockerham CC (1984) Estimating F-statistics for the analysis of population structure. Evolution 38: 1358-1370.

7. Goudet J (2001) FSTAT, a program to estimate and test gene diversities and fixation indices (version 2.9.3). <http://www.unil.ch/izea/softwares/fstat.html>.

8. Allendorf FW, Phelps SR (1981) Use of allele frequencies to describe population genetic structure. Canadian Journal of Fisheries and Aquatic Sciences 38: 1507-1514.

9. Blank WA, Reis ER, Thiong'o FW, Braghiroli JF, Santos JM, et al. (2009) Analysis of *Schistosoma mansoni* population structure using total fecal egg sampling. Journal of Parasitology 95: 881-889.
