# Supplementary material for: Non-Invasive Sampling of Schistosomes from Humans Requires Correcting for Family Structure
Source: PLoS Negl Trop Dis. 2013 Sep 19;7(9):e2456. doi: 10.1371/journal.pntd.0002456 (PMC3777896; doi:10.1371/journal.pntd.0002456)
Supplement: Supporting Information S2 — Performance of colony using monogamous and polygamous mating system designation. (DOCX) [file pntd.0002456.s002.docx]

**Performance of Colony Using Monogamous and Polygamous Mating System Designation**

COLONY v.2.0 [1,2] was used to partition individual miracidia into their probable sibling groups. We performed analyses with two different user-defined options, first using a monogamous mating system, and second using a polygamous mating system (to include half-sibships generated from matings of genetic clones). We found that the analyses using the polygamous mating system resulted in the identification of more full-sibling families (Fig. S2). Because the analyses using a “monogamous” mating system correctly assigned offspring to their families 99% of the time (97%, 100%, and 100% for the three datasets analyzed), it appears that COLONY tends to split full sibling families when the “Polygamy” option is chosen.


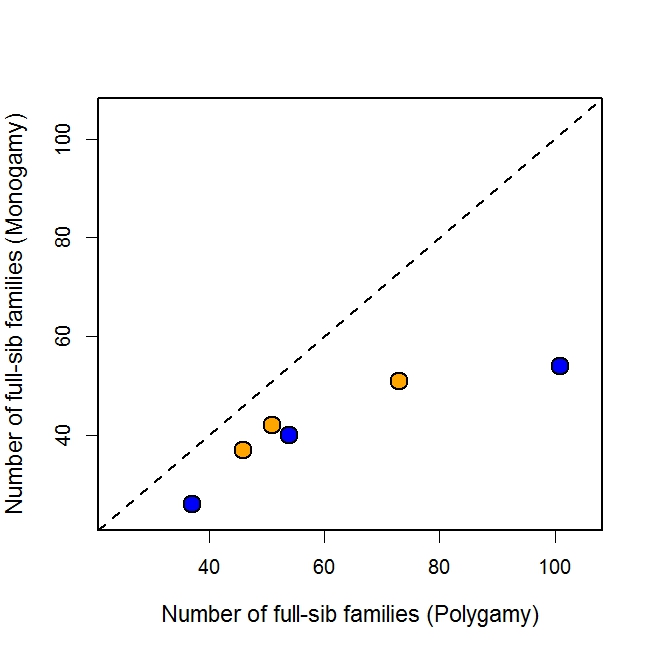


**Figure S2**. Comparison of the total number of full sibling families identified by COLONY v. 2.0 in samples of schistosome miracidia using two different user defined options: mating system monogamous or polygamous. Blue circles represent results from Kenyan patients and orange circles represent results from simulated data sets. Note that in all cases, fewer full sibling families were identified with analyses designated as “monogamous” compared to “polygamous”.

**References**

1. Wang J, Santure AW (2009) Parentage and sibship inference from multilocus genotype data under polygamy. Genetics 181: 1579-1594.

2. Jones OR, Wang JL (2010) COLONY: a program for parentage and sibship inference from multilocus genotype data. Molecular Ecology Resources 10: 551-555.
