# Supplementary material for: Non-Invasive Sampling of Schistosomes from Humans Requires Correcting for Family Structure
Source: PLoS Negl Trop Dis. 2013 Sep 19;7(9):e2456. doi: 10.1371/journal.pntd.0002456 (PMC3777896; doi:10.1371/journal.pntd.0002456)
Supplement: Supporting Information S3 — Performance of alternative methods of sibship reconstruction. (DOCX) [file pntd.0002456.s003.docx]

**Performance of alternative methods of sibship reconstruction**

We used an alternative software package, PEDIGREE [1] to identify sibling structure within our samples. The PEDIGREE analysis included 5,000,000 iterations of the Markov Chain for each of 10 replicates (weight=6 and the temperature for 5 replicates was 10, and 20 for the remaining). Comparisons between the results from COLONY (as reported in the manuscript) and PEDIGREE were nearly identical with respect to the number of full sibling families (Pearson’s correlation: r = 0.991, P < 0.0001), (Fig. S1), the variance to mean ratios (VMR: r = 0.9325, P < 0.0001), and the percentage of miracidia belonging to a family of four or more individuals (%: r = 0.972, P <0.001) (Fig. S1).

**Figure S1**. Comparison of the output from COLONY and PEDIGREE for samples of schistosome miracidia collected from 12 patients. A. The number of Full sibling families (FSF), B. The variance to mean ratios of reproductive output among families (VMR), and C. The percentage of miracidia belonging to a family of 4 or more (% With Family). Lines indicate a perfect one-to-one correlation.

**References**

1. Smith BR, Herbinger CM, Merry HR (2001) Accurate partition of individuals into full-sib families from genetic data without parental information. Genetics 158: 1329-1338.
