# Supplementary material for: Non-Invasive Sampling of Schistosomes from Humans Requires Correcting for Family Structure
Source: PLoS Negl Trop Dis. 2013 Sep 19;7(9):e2456. doi: 10.1371/journal.pntd.0002456 (PMC3777896; doi:10.1371/journal.pntd.0002456)
Supplement: Supporting Information S4 — Assessing the variance in the “corrected” one-per-family datasets. (DOCX) [file pntd.0002456.s004.docx]

**Assessing the variance in the “corrected” one-per-family datasets**

To remove family structure from the datasets, we recommend resampling the dataset to include only one member from each family in the dataset. We also recommend resampling the dataset a large number of times because the range of values one could obtain is quite large if only a single one-per-family dataset is used (Fig. S3).

**Figure S3**. Illustration of the range of values that were obtained by creating only a single one-per-family dataset (see main text for details). The plot shows pairwise F_ST_ values between schistosome infrapopulations of 12 human patients. Blue circles show the pairwise F_ST_ values of the raw, uncorrected datasets and green circles show the mean F_ST_ values of 1000 resampled datasets that include only one member of each family. Dotted lines show the range of pairwise F_ST_ values that were obtained from the one-per-family datasets. Note the large range of values, although most are smaller than the values from the raw datasets (red circles indicate the exceptions). The 95% confidence intervals that surround the mean of the 1000 samples are too small to plot (i.e., 1000 samples resulted in high accuracy and precision).
