# Supplementary material for: Non-Invasive Sampling of Schistosomes from Humans Requires Correcting for Family Structure
Source: PLoS Negl Trop Dis. 2013 Sep 19;7(9):e2456. doi: 10.1371/journal.pntd.0002456 (PMC3777896; doi:10.1371/journal.pntd.0002456)
Supplement: Supporting Information S5 — Temporal sampling: Examining the genetic distribution of miracidia obtained from temporally collected fecal samples. (DOCX) [file pntd.0002456.s005.docx]

**Temporal sampling: examining the genetic distribution of miracidia obtained from temporally collected fecal samples**

As described in the manuscript, temporal fecal samples were obtained for three patients. We found that different families were represented on different days. Figure S4 graphically displays the membership of each family collected in each fecal sample.

C..


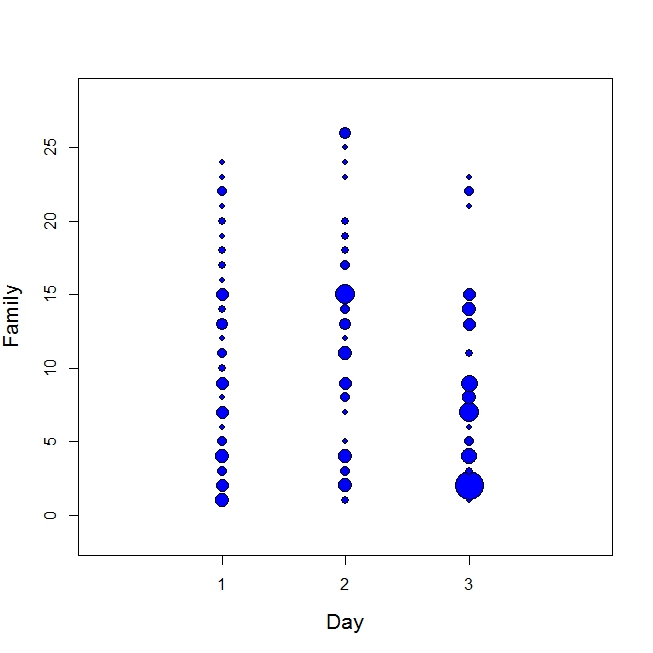

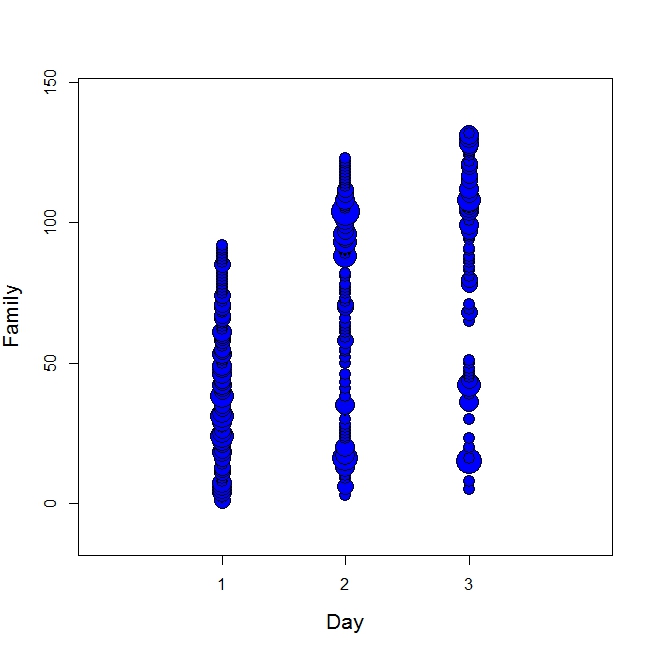

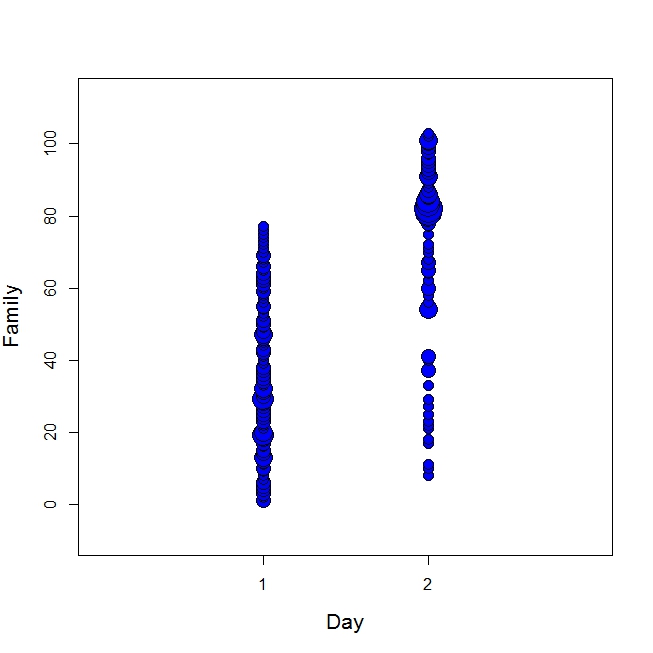


B.

**Figure S4**. Family representation of the schistosome miracidia collected from multiple different fecal samples from the same patient. Size of circle indicates the relative number of individuals per family. Note the different representation on each of the days, which in some cases leads to genetic differentiation between samples collected on different days. A. Patient 2, B. Patient 3, and C. Patient 12.

A.

A.
